# Intestinal Stem Cell Niche Insights Gathered from Both *In Vivo* and Novel *In Vitro* Models

**DOI:** 10.1155/2017/8387297

**Published:** 2017-09-07

**Authors:** Nikolce Gjorevski, Paloma Ordóñez-Morán

**Affiliations:** ^1^Laboratory of Stem Cell Bioengineering, Institute of Bioengineering, School of Life Sciences (SV) and School of Engineering (ST), École Polytechnique Fédérale de Lausanne (EPFL), Lausanne, Switzerland; ^2^Swiss Institute for Experimental Cancer Research (ISREC), École Polytechnique Fédérale de Lausanne (EPFL), Lausanne, Switzerland

## Abstract

Intestinal stem cells are located at the base of the crypts and are surrounded by a complex structure called niche. This environment is composed mainly of epithelial cells and stroma which provides signals that govern cell maintenance, proliferation, and differentiation. Understanding how the niche regulates stem cell fate by controlling developmental signaling pathways will help us to define how stem cells choose between self-renewal and differentiation and how they maintain their undifferentiated state. Tractable *in vitro* assay systems, which reflect the complexity of the *in vivo* situation but provide higher level of control, would likely be crucial in identifying new players and mechanisms controlling stem cell function. Knowledge of the intestinal stem cell niche gathered from both *in vivo* and novel *in vitro* models may help us improve therapies for tumorigenesis and intestinal damage and make autologous intestinal transplants a feasible clinical practice.

## 1. Introduction

The intestine represents the most vigorously renewing adult tissue, which undergoes rapid turnover in order to prevent damage from stress factors; its tissue-specific stem cells are essential for tissue homeostasis in the adult organism [1]. These undifferentiated cells residing at the bottom of the crypts of Lieberkühn are able to produce a large number of differentiated progeny as well as to self-renewal. Due to their relevant function, many efforts have been done in the last years to define the exact localization of the intestinal stem cells and its properties. There is now evidence that at least two types of stem cells coexist in the small intestine. Best characterized are the leucine-rich-repeat-containing G-protein-coupled receptor 5-expressing (Lgr5^+^) stem cells which divide approximately every 24 hours, and they are interspersed between the terminally differentiated Paneth cells [2]. The *Lgr5* gene was selected from a panel of intestinal Wnt targets for its restricted crypt expression (columnar base cells, CBC) and was identified as a marker gene of stem cells in the small intestine and colon [2]. Very recent findings have found that Lgr5^+^ stem cell population is not homogenous. The expression of the RNA-binding protein Mex3a labels a slowly cycling subpopulation of Lgr5^+^ ISCs that contribute to all intestinal lineages. Thus, Mex3a defines a reserve-like ISC population within the Lgr5^+^ compartment [3]. The second type of stem cells are located at the +4 position of the intestinal crypt and are called label-retaining cells (LRCs) as they show long-term label retention upon irradiation damage and pulse labeling with BrdU. These cells remain quiescent and act as a reserve population that can give rise to all intestinal cell lineages after tissue damage [4–8]. Some reports point out that there is an apparent dichotomy between quiescent versus cycling stem cells that in fact reflect a continuum of phenotypes dictated by different thresholds of expression of key regulators (e.g., signals and/or transcription factors) that modulate stem-like functions [7, 9–13]. Future experiments for a better identification of these mechanisms and the features of the +4 LRC stem cell populations are still needed in order to understand the capacity of the intestinal tissue to induce a regenerative response under (radiation induced) tissue injury. In this review, we will mostly focus on the *in vivo* and *in vitro* models for intestinal CBC stem cell niche.

**Figure 1:**
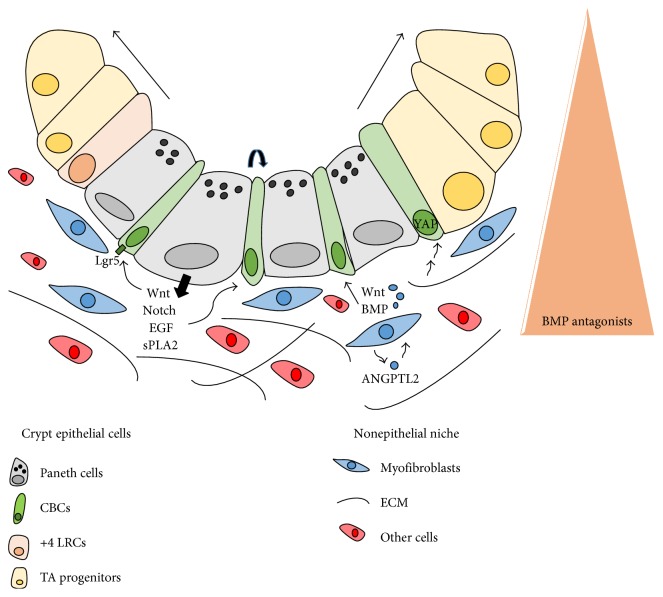
The stem cell niche of the small intestine. Epithelial and nonepithelial environments support the signals required for stem cell maintenance. Among them, Wnt and Notch signaling have been defined as major determinants for stem cell self-renewal, for proliferation/differentiation of stem cells in the crypt. Stromal BMP antagonists regulate the crypt-villus axis, and the extracellular cell matrix (ECM) support signals that control stem cell fate. Other cells: neural, immune, and endothelial cells. TA: transit-amplifying progenitors; sPLA2: secreted phospholipases A2.

Control of proliferation, self-renewal, and lineage specification of the stem cells in the crypt are believed to be directed by an actively regulated process based on cell-cell and cell-stroma interactions [14]. The ISC niche or microenvironment is composed of epithelial and underlying nonepithelial cells within the lamina propia populated by stromal, immune, endothelial, and neural cells that support paracrine and/or autocrine signaling (Figure 1). The ISC niche also comprises the extracellular matrix (ECM), a highly dynamic structure that continuously undergoes controlled remodelling, mediated by metalloproteinases that are responsible for ECM degradation [15]. The ECM interacts with the different cells in the niche to regulate stem cell fate [16] (Figure 1). Overall, the components of the niche tightly modulate Wnt, Notch, epidermal growth factor (EGF), bone morphogenic protein (BMP)/transforming growth factor (TGF) *β*, and Hedgehog signaling pathways to maintain proliferation/differentiation balance [17–19].

Functional analysis of stem cells and their environment has been hampered by a lack of suitable *in vitro* systems allowing long-term culture and until some years ago, the only possible strategy to analyse such interactions for a potential role in intestinal development, homeostasis, damage or tumorigenesis was the time-consuming tissue-specific mouse models. For example, *Achaete-scute complex homolog 2* (Ascl2) was reported to be responsible for controlling intestinal stem cell fate by using transgenic mice [20]. In 2009, two groups developed a three-dimensional (3D) culture model of freshly isolated crypt cells from murine small intestine and colon [21–23], and later this method was set up for human samples [24, 25]. These assays maintain basic crypt-villus physiology and permit long-term intestinal epithelial expansion as sphere-like organoids. The stem cells are embedded in Matrigel, a gelatinous protein mixture secreted by mouse sarcoma cells containing structural proteins such as laminin, entactin, and collagen in combination with several growth stimuli essential for crypt proliferation (the Wnt agonist R-spondin1, EGF, and the BMP inhibitor Noggin). Single-sorted Lgr5^+^ stem cells are sufficient to give rise to organoids in culture which contain all differentiated lineages: Paneth cells at the base of the crypt and enteroendocrine, goblet cells, and enterocytes that migrate upwards the villus. Importantly, these cultures allow ex vivo monitoring intestinal stem cell function with respect to self-renewal and production of rapidly dividing crypt progenitor cells and differentiated lineages and are therefore comparable to the *in vivo* situation [21]. In this review, we will compare *in vivo* models to the most novel *in vitro* technology which will improve in the next years our understanding of stem cell behavior.

## 2. *In Vivo* Models of the Stem Cell Niche

### 2.1. CBC Epithelial Niche

Stem cells require the support of neighbouring epithelial cells to maintain their function. The epithelial niche modulates several signaling cascades, the Wnt pathway being one of the main regulators of stem cell self-renewal. Genetic deletion of the Wnt pathway's main players (*β*-catenin, Tcf4 knockout models) or ectopic expression of the secreted Wnt antagonist Dickkopf-1 (Dkk1) disrupts intestinal epithelial homeostasis, leading to crypt loss, reduced proliferation, and altered differentiation [26–28]. Similarly, overactivation of the Wnt pathway in mice, by overexpression of the Wnt agonists roof plate-specific spondin 1 (R-spondin1) and R-spondin3 or by deleting *Adenomatous polyposis coli (Apc)*, drives hyperplasia and increases the expansion of intestinal stem cell niche [29–31]. The Wnt target genes, *EphB2*, *EphB3*, and their ligands ephrins, are key coordinators of migration and proliferation in the stem cell niche. EphB knockouts show that these proteins determine cell positioning along the crypt-villus axis in the intestinal epithelium [32]. Furthermore, EphB signaling promotes cell-cycle reentry of progenitor cells and contributes to the mitogenic activity in the adult mouse small intestine and colon [33].

“What is the main source of epithelial Wnt signals within the intestine?” The secretory Paneth cells adjacent to CBCs secrete Wnt3, and they constitute an important part of the small intestinal stem cell niche. They are known to produce bactericidal products such as lysozyme and cryptdins/defensins, and in addition, they can also produce TGF*α*, Notch (Dll4), and EGF factors that regulate stem cell maintenance [34, 35]. Reduction of number of Paneth cells in Gfi1^−/−^ mouse model, transgenic expression of diphtheria toxin A under the Paneth cell-specific cryptdin 2 promoter (*CR2-tox176*), and conditional deletion of *Sox9* showed that the stem cells were coincidently decreased in number [34]. Some studies indicate that these cells are dispensable for small intestinal homeostasis; however, it should be determined in additional mouse models able to also achieve a total disruption of Paneth cells [36]. Although, this exact type of cells are not present in the colon, Clarke's group found goblet cells interdigitated with Lgr5^+^ stem cells that contained a distinct cKit/CD117^+^ crypt base subpopulation which expressed the Notch ligands Delta-like (Dll) 1, Dll4, and EGF. *In vivo*, this colonic cKit population was regulated by Notch signaling [37]. Later on, Clevers lab described equivalent cells called regenerating islet-derived family member (Reg) 4^+^-expressing deep crypt secretory (DCS) cells (called Paneth/goblet-like cells) that are intermingled with the Lgr5^+^ colonic stem cells at the base of the crypt. These cells also produce Wnt and Notch factors to support essential growth and maintenance signals. In these mouse experiments, the ablation of these types of cells resulted in loss of stem cell function and disruption of colon homeostasis [38]. When Paneth or DCS cells were sorted together with Lgr5^+^ cells, the signals provided by them markedly increased differentiation and organoid growth from a single stem cell in the *in vitro* culture [34, 38]. Novel data have revealed that Paneth and DCS cells are also secreting the phospholipases A2 (sPLA2s) which inhibit Wnt pathway through intracellular activation of Yap1. Importantly, this cascade affects stem cell niche during homeostasis [39].

Several Notch mouse models evidence the impact of this pathway in epithelial stem cell niche. Indeed, Lgr5^+^ stem cells are critically dependent on Notch, which depend on direct cell-cell contact as the Paneth cells are the main sources of Notch signals [40–46]. This is the case of simultaneous inactivation of Dll1 and Dll4 which resulted in the complete conversion of proliferating progenitors into postmitotic goblet cells, concomitant with the loss of Lgr5^+^ SCs [41]. A negative regulatory mechanism of Wnt and Notch influencing intestinal stem cells in the gut was nicely shown by Tian et al. When Notch pathway was blocked, it perturbed intestinal stem cell function by causing a derepression of the Wnt pathway, leading to misexpression of prosecretory genes. Then, attenuation of Wnt rescued the phenotype associated with Notch blockade [43].

Other studies show *Leucine-rich repeats and immunoglobulin-like domains (Lrig) 1*, a direct Myc target gene as part of a negative feedback loop that modulates the proliferation of intestinal progenitor cells. *Lrig* knockout mouse induces upregulation of EGFR, ErbB2, and ErbB3 promoting downstream activation of c-Myc within intestinal stem and progenitor cells [17, 47]. The EGF pathway affects stem cell function by regulating the phosphoinositide 3-kinase (PI3K), mitogen-activated protein kinase (MAPK), and/or protein kinase C (PKC) pathways among other cascades [48]. These and further studies on this direction may lead to next-generation stem cell-based therapies.

### 2.2. CBC Nonepithelial Niche

Vigorous crosstalk between the epithelium and the underlying nonepithelial niche is required to define the crypt-villus axis. It is well established that mesenchymal cells secrete BMP antagonists such as Gremlin 1 and Gremlin 2 at the bottom of the crypts which supports compartmentalization [49]. Thus, BMP signaling is inhibited for a right intestinal epithelial renewal. Indeed, mouse transgenic overexpression of the BMP antagonist Noggin affects crypt expansion and increased stem cell numbers [50, 51]. Moreover, genetic models carrying BMPR1A inactivation or deficiency of its downstream effector PTEN show an inhibition of BMP signaling that enhances AKT activation and an increase in Wnt signaling [1, 50]. Hedgehog signaling is also involved in this crosstalk by modulating stromal BMPs [52]. The gradient of Wnt and BMP pathway by diffusion of ligands along the crypt acts as a balance of cell differentiation/proliferation. Wnt is higher at the crypt base, whereas BMP pathway, which inhibits proliferation, has an opposite pattern of expression [1] (Figure 1).

Mesenchymal cells also secrete Wnt proteins, and R-spondins have been detected in the intestinal stroma [19, 53–55]. Experiments by using inducible mouse deletion (only epithelial cells) of Porcupine (an endoplasmic reticulum resident O-acyltransferase essential for the secretion and activity of all Wnts) showed that the cells had normal proliferation and differentiation, indicating that epithelial Wnt is dispensable for stem cell maintenance. Then, it was observed that intestinal stromal cells endogenously expressing Wnts and R-spondin3 support the growth of Porcupine-deficient organoids ex vivo, pointing out that stromal production of Wnts can fully support murine intestinal homeostasis [56].

All these data suggest that Wnt signals from Paneth cells can be replaced by stromal ones, so nonepithelial activation of Wnt pathway may support intestinal stem cell maintenance. On this direction, recent studies show that a subpopulation of mesenchymal cells marked by the winged-helix transcription factor Foxl1 is critical in maintaining stem cells. These cells produce Wnt factors, and their ablation reduces crypt growth. However, there is a need to better identify this subset of mesenchymal cells [57]. In addition, the autocrine secretion of ANGPTL2 by subepithelial myofibroblasts affects BMP production which then modulates intestinal organoid growth and size. Moreover, intestinal damage of *Angptl2* knockout mice reduces CBCs and influences Wnt pathway; however, ANGPTL2 is dispensable for intestinal homeostasis [58] (Figure 1).

Remarkably, some other cell populations in the niche (immune, endothelial, and neural cells) also influence the stem cell behaviour by modulating the different signaling cascades. They secrete growth factors, cytokines, and ligands that alter the stem cell fate [19, 59]. Upcoming work is required to reveal a more detailed comprehension of the interplay and components of this complex cellular network.

## 3. *In Vitro* Models of the ISC Niche

### 3.1. Intestinal Organoids

Animal models have provided invaluable insight into the nature and hallmarks of the intestinal stem cell, as well as the set of microenvironmental inputs that govern its behavior and constitute the intestinal stem cell (ISC) niche. However, in addition to ethical and practical considerations, animal-based studies suffer multiple limitations in the scope of scientific questions they can address. In particular, mouse models generally do not afford the dynamic and multifactorial observation and control that are required for securing comprehensive understanding of ISCs and their niche. Further, whereas the mouse intestine is an adequate approximation of its human counterpart, several crucial developmental and histological differences exist [60], and mouse-based studies may fall short in providing insight that is also relevant for humans. *In vitro* models of the ISC niche circumvent these problems by offering a level of accessibility and tractability that is difficult or impossible to achieve *in vivo*.

Driven by both basic research and therapeutic objectives, researchers have cultured stem cells *in vitro* for several decades. A decade ago, Sasai demonstrated that, aside from directing pluripotent stem cells to commit toward a certain lineage, thus obtaining populations of differentiated cells, stem cells and their progeny can follow their innate developmental programs and self-organize into structures that mimic multiple histological and functional aspects of real organs [61, 62]. These organ mimetics generated *in vitro* were termed organoids.

Intestinal organoids, or “miniguts”, generated in the laboratory of Hans Clevers, were among the first types of stem cell-derived organoids reported [21, 24]. Sato et al. showed that intestinal crypts or single-dissociated Lgr5-expressing ISCs embedded in Matrigel and provided with niche signals, including R-spondin1, Noggin, and EGF, not only survive and proliferate but also undergo morphogenesis and differentiation to produce structures that approximate the adult intestine: crypt-like projections radiate outward from a spherical epithelial structure that surrounds a central lumen. In addition to cycling ISCs, housed at the proximal ends of the crypt-like buds, intestinal organoids contain all differentiated intestinal cell types, which are represented at the ratios found in the native intestine [63] and in spatial arrangements that closely mimic the patterning of the crypt-villus axis. Importantly, these structures reconstitute the principal geometric, architectural, and cellular hallmarks of the ISC niche—Lgr5-expressing ISCs are attached to a basement membrane-like hydrogel. ISCs and Paneth cells are represented in numbers and ratios reflecting those *in vivo*, thus forming a bud structure of similar in shape and size to those of the intestinal crypt. Small modifications of the culture protocol—notably, the addition of Wnt3a—allow for the culture of adult human ISC-derived intestinal organoids [24]. It should be emphasized, however, that these structures feature a round, cystic architecture, thus missing the crypt-like domains of mouse intestinal organoids.

In addition to adult ISCs, intestinal organoids have been generated from induced pluripotent stem cells (PSCs), using a protocol inspired by human embryonic development [64]. PSCs were first treated with activin A to induce the formation of definitive endoderm, which was then steered toward mid/hindgut fates by treatment with FGF4 and Wnt3a. Culturing of the resulting mid/hindgut spheroids in Matrigel, under conditions used for the culture of adult ISCs and crypts [21], gave rise to intestinal organoids. Notably, PSC-derived human organoids are organized into crypt- and villus-like domains, contain the major differentiated epithelial cell types, and, interestingly, are enveloped by a sheath of mesenchyme, comprised of myofibroblasts and smooth muscle cells, thus recapitulating an additional aspect of the ISC niche.

Aside from promising to revolutionize basic and clinical research, by serving as models of development and disease, platforms for drug discovery and toxicity screens and sources of tissue for cell-based therapies [23, 65], intestinal organoids complement *in vivo* studies in our quest to dissect the ISC niche and define the mechanisms whereby it exerts its influence on stem cells. Indeed, to demonstrate that Paneth cells constitute the ISC niche, as discussed above, mouse models were used in conjunction with intestinal organoids [34, 66]. Likewise, organoids have been instrumental in elucidating the roles of various genes, including R-spondin and Lgr4/5 [67, 68] and YAP [69, 70] in the regulation of ISC fate.

The format of common organoid culture models allows for relatively easy and routine manipulations of the soluble microenvironment of ISCs. Beyond a set of soluble cues, the stem cell niche also comprises adhesion and mechanical signals from the surrounding ECM [71], which are likely to be as important as morphogens and growth factors in regulating ISC fate [72–75]. Typical intestinal organoid models, however, employ Matrigel—an ECM protein-rich hydrogel derived from the Engelbreth-Holm-Swarm sarcoma—as the 3D matrix. Matrigel, while clearly providing essential adhesive and mechanical cues, without which organoid formation would not be possible, remains a black box in regard to its contribution to the ISC niche. That is, Matrigel is a complex multicomponent mixture with ill-fined and variable biochemical and biophysical properties [76, 77] and the specific components and mechanisms whereby this material influences ISC fate are unclear. In the following sections, we will discuss recent advances in using biomaterials and bioengineering approaches to overcome the limitations of Matrigel, secure a more holistic understanding, and introduce additional levels of control over the ISC niche.

### 3.2. Toward a Synthetic ISC Niche: Using Synthetic Matrices to Deconstruct the Native Intestinal ECM

Synthetic hydrogels, comprising a water-swelled polymer network, can be rendered biocompatible and biofunctional through the incorporation of essential biological signals and used as well-defined alternatives to animal-derived ECM gels, such as collagen and Matrigel [78–80]. Moreover, these materials provide a biologically “blank” 3D environment into which biochemical and biophysical factors found in native tissues can be introduced and varied in a systematic and controlled manner, thus interrogating their cellular effects and evaluating them as potential stem cell niche components.

**Figure 2:**
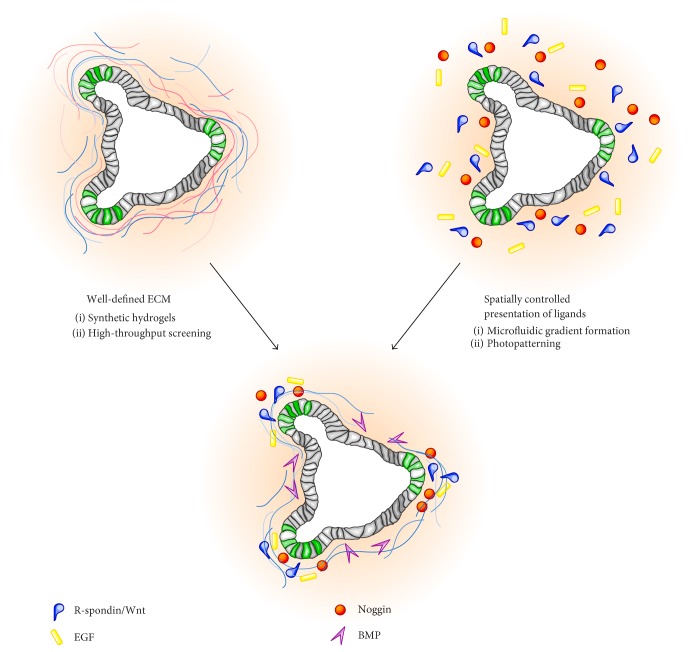
Engineering the ISC niche *in vitro*. Bioengineering approaches could further increase the tractability of organoid models and their fidelity to the real intestine. Synthetic matrices simplify the complexity of Matrigel and offer a powerful new toolkit with which to examine the effects of individual or combinations of ECM and mechanical niche signals. Microengineering approaches can be used to introduce spatial and temporal control over the biochemical and biophysical environment of ISCs, thereby mimicking the native niche more closely.

We recently took advantage of poly(ethylene glycol) (PEG) hydrogels to identify ECM components that control ISC fate and used this knowledge to construct well-defined and tunable matrices for the culture of ISCs and intestinal organoids [81] (Figure 2). Inspired by their localization to the basement membrane of mouse and human intestinal crypts *in vivo* [60, 74, 82–85], we assessed the effect of laminin-111, collagen-IV, fibronectin, hyaluronic acid, and perlecan on ISC self-renewal, differentiation, and organoid formation in the context of a 3D PEG hydrogel. We found that all components enhanced ISC survival and colony formation; laminin-111, collagen-IV, and fibronectin displayed the strongest positive effects. Notably, the fibronectin-derived RGD (Arg-Gly-Asp) peptide was sufficient in supporting ISC expansion in synthetic matrices. On the other hand, laminin-111 was of crucial importance for the concerted cycles of ISC self-renewal, differentiation, and morphogenesis that drive organoid formation; none of the other ECM components tested were even minimally effective. Although informative, our study examined the effects of only a handful of ECM components found *in vivo*. Future studies not only could take a system-level approach and expanded the number of ECM factors tested but also investigate potential interactive effects of multiple components. Sophisticated high-throughput approaches to generate and analyze multifactorial environments, which have already been used to study other stem cell systems [86, 87], seem ideally suited for further deconstructing the complexity of the ISC niche.

In addition to soluble and tethered molecular factors, ISCs *in vivo* experience physical signals from the microenvironment, including the mechanical properties of their surrounding ECM. The mechanical environment is now recognized as a major extrinsic regulator of multiple stem cell systems [88]. Our understanding of potential physical regulators of ISC fate is minimal, owing to the difficulty of performing controlled mechanical perturbations in both mouse models and Matrigel-based organoid culture. Nevertheless, recent *in vivo* studies provide clues that mechanical forces may directly control ISC proliferation in the colon [89]. We used the mechanically tunable PEG matrices to examine the effect of matrix mechanical properties on ISC expansion and organoid formation [81] and observed profound effects. In particular, we found that relatively stiff (shear modulus of ~1 kPa) matrices were optimal for ISC expansion, whereas ISC survival and colony formation in soft matrices was exceedingly low. In contrast, ISC differentiation and organoid formation were impaired by stiff matrices and only occurred in soft ones. We again took advantage of the versatility of the PEG hydrogel system to shed light on the molecular mechanisms underlying the mechanical effects on ISC expansion and organoid formation, which, surprisingly also accounted for the seemingly contradictory influence of stiffness on these two processes. In particular, we found that the matrix mechanics regulates ISC behavior by controlling the activity of Yes-associated protein 1 (YAP), which is a known mechanotransducer in other cellular systems [90, 91], and is also required for ISC expansion and organoid formation [69, 70, 81]. We showed that stiff matrices enhance ISC colony formation by inducing nuclear translocation of YAP in single-embedded ISCs. However, continued ISC proliferation within a stiff environment led to cell confinement and compression, which in turn resulted in gradual YAP inactivation. Creating matrices that are initially stiff enough to induce YAP activation but soften in a controlled manner to prevent cell compression rescued YAP inactivation and supported both ISC expansion and organoid formation. Thus, we used modular PEG-based matrices to unveil matrix stiffness as another important component of the ISC niche [81].

### 3.3. Future Perspective: Using Microengineering to Establish a Homeostatic ISC Niche *In Vitro*

A key difference between the intestinal niche *in vivo* and current organoid models is not only the types of niche components available but also the mode in which they are presented. Current organoid cultures contain essential niche signals, including Wnt and Notch pathway agonists, and BMP inhibitors. However, these soluble components are included in the cell culture medium, wherein they eventually reach a uniform concentration. In contrast, master regulators of intestinal biology *in vivo* are presented in distinct spatiotemporal patterns, which are crucial for the regionalization of the intestine and the establishment of the ISC niche. For example, Wnt signals *in vivo* are produced by the subepithelial mesenchyme and Paneth cell and thus restricted to the bottom of the crypts where they are crucial for maintaining ISCs in a self-renewing state [92]. Bone morphogenetic protein (BMP) and Sonic Hedgehog (Shh) signals, are, on the other hand, enriched in the villus region, where they suppress proliferation and ensure differentiation into functional enterocytes [92]. This difference in the presentation of soluble cues may account for the fact that intestinal organoids are continuously expanding structures, with new crypt-like buds forming perpetually. Thus, intestinal organoids currently mimic a developmental or regenerative, rather than a homeostatic state, wherein ISC self-renewal is balanced by differentiation and apoptosis to establish a stable niche (the intestinal crypt).

Bioengineers have developed a number of strategies for controlling the spatiotemporal patterns of soluble and tethered cues in soft 3D media, similar to the matrices required for organoid formation. Microfluidically generated morphogen gradients are perhaps the most widespread approach. Here, 3D hydrogels are microstructured using lithographic [93–95] or ablative [96] techniques. The resulting channels are loaded with a molecular of interest, which forms a gradient through the surrounding permeable gel. The shape of the gradient and, thus, the spatiotemporal mode of biomolecule delivery to encapsulated cells can be controlled by varying the concentration of the molecule at the source (the channel), the flow rate, and the diffusive properties of the permeable medium. Photopatterning approaches provide even finer spatial and temporal control over the distribution of mechanical and tethered molecular cues in 3D gels. These strategies use controlled illumination to locally change the properties of hydrogels that have been engineered to contain photosensitive building blocks [97–99]. The molecular changes induced can then be used to add or remove molecules or alter the mechanical stiffness of a desired region, at a desired time. We believe that these and other approaches for controlling the spatial and temporal presentation of diffusible or immobilized cues may be useful for the generation of the separate molecular zones seen in the native intestine, which could in turn contribute toward creating a more realistic *in vitro* model of a stable, homeostatic ISC niche (Figure 2).
